# Prenylated flavonoid morusin protects against TNBS-induced colitis in rats

**DOI:** 10.1371/journal.pone.0182464

**Published:** 2017-08-10

**Authors:** Zora Vochyánová, Marie Pokorná, Dominik Rotrekl, Václav Smékal, Petr Fictum, Pavel Suchý, Jan Gajdziok, Karel Šmejkal, Jan Hošek

**Affiliations:** 1 Department of Molecular Biology and Pharmaceutical Biotechnology, Faculty of Pharmacy, University of Veterinary and Pharmaceutical Sciences Brno, Brno, Czech Republic; 2 Department of Natural Drugs, Faculty of Pharmacy, University of Veterinary and Pharmaceutical Sciences Brno, Brno, Czech Republic; 3 Department of Pathological Morphology and Parasitology, Faculty of Veterinary Medicine, University of Veterinary and Pharmaceutical Sciences Brno, Brno, Czech Republic; 4 Department of Human Pharmacology and Toxicology, Faculty of Pharmacy, University of Veterinary and Pharmaceutical Sciences Brno, Brno, Czech Republic; 5 Department of Pharmaceutics, Faculty of Pharmacy, University of Veterinary and Pharmaceutical Sciences Brno, Brno, Czech Republic; University of South Carolina School of Medicine, UNITED STATES

## Abstract

Morusin is a prenylated flavonoid isolated from the root bark of *Morus alba*. Many studies have shown the ability of flavonoids to act as anti-inflammatory agents. The aim of this study was to evaluate the effect of morusin on experimentally colitis induced by 2,4,6-trinitrobenzensulfonic acid in Wistar rats and to compare it with sulfasalazine, a drug conventionally used in the treatment of inflammatory bowel disease. Morusin was administered by gavage at doses of 12.5, 25, or 50 mg/kg/day for five days. The colonic tissue was evaluated macroscopically, histologically, and by performing immunodetection and zymographic analysis to determine the levels of antioxidant enzymes [superoxide dismutase (SOD) and catalase (CAT)], interleukin (IL)-1β, and transforming growth factor (TGF)-β1 and the activities of matrix metalloproteinases (MMP) 2 and 9. The tissue damage scores were significantly reduced with increasing dose of morusin, however efficacy was not demonstrated at the highest dose. At the dose of 12.5 mg/kg, morusin exerted therapeutic effectivity similar to that of sulfasalazine (50 mg/kg). This was associated with significant reduction of TGF-β1 levels and MMP2 and MMP9 activities, and slight reduction of IL-1β. Our results suggest that morusin possesses therapeutic potential for the treatment of chronic inflammatory diseases.

## Introduction

Flavonoids are a class of secondary plant metabolites showing a wide spectrum of biological activities, of which the anti-inflammatory and antioxidant effects draw the most attention. The arrangement of functional groups on the flavonoid skeleton necessary for enhancement of the anti-inflammatory activity has been reported previously [1]. Generally, the presence of a double bond between C-2 and C-3 is essential for the anti-inflammatory activity and it appears to be required for the inhibition of phospholipase A2 and 5- and 12-lipoxygenases [2]. Another study showed that the presence of a carbonyl function group at position C-4 was important for the optimal inhibition of the expression of the tumor necrosis factor (TNF)-α induced intercellular adhesion molecule (ICAM)-1 [3].

Prenylated flavonoids represent an interesting subgroup of flavonoids because they combine the hydrophilic properties of the basic flavonoid skeleton with lipophilic side chains of C5 isoprene units possessing different lengths and modifications. Prenylation may increase bioactivity by accelerating absorption, reducing cell efflux, and enhancing the affinity for biological structures [4]. These features can make prenylated flavonoids leading compounds for the treatment of chronic inflammatory diseases, such as inflammatory bowel disease (IBD) or rheumatoid arthritis.

**Fig 1:**
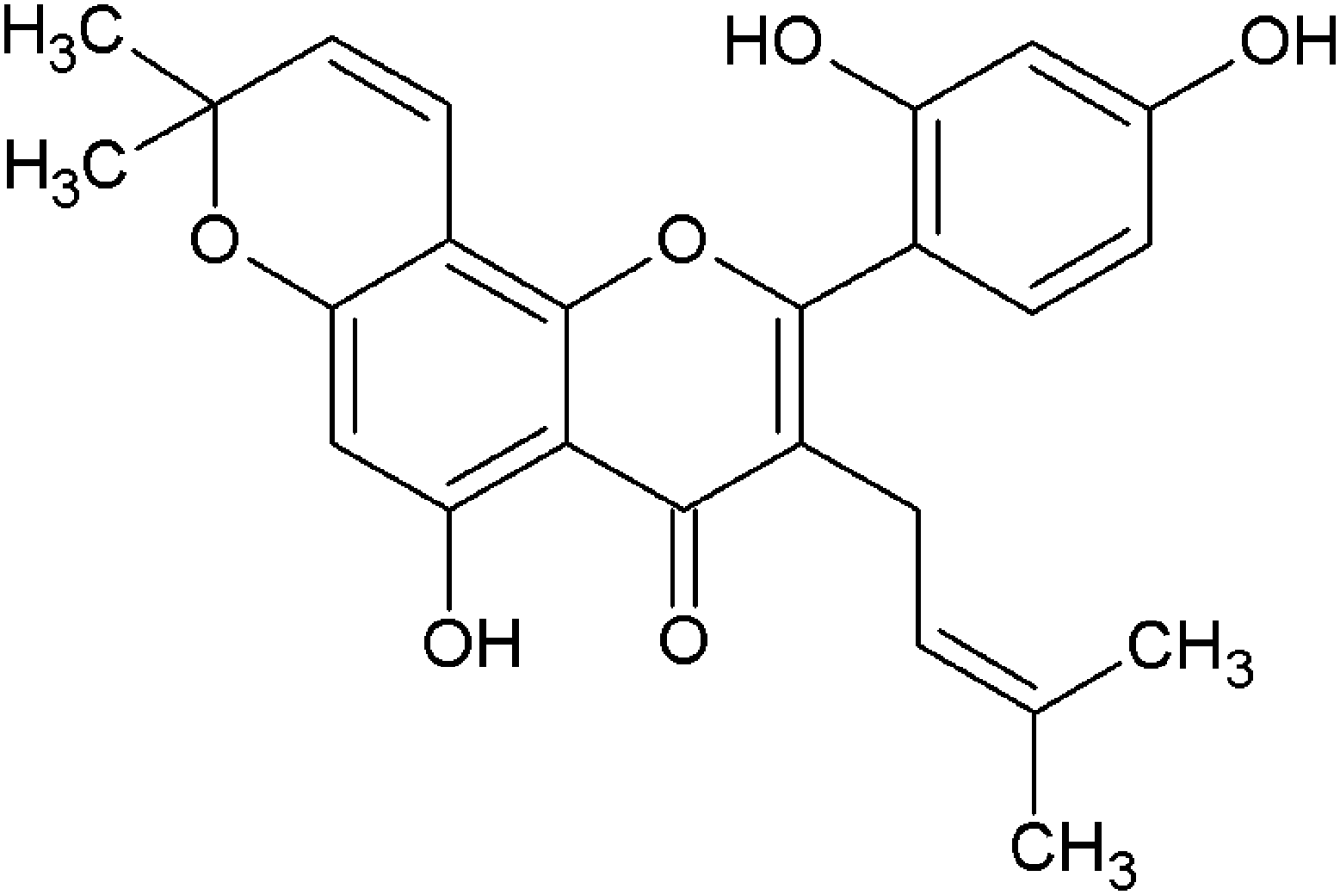
Chemical structure of morusin.

Numerous prenylated flavonoids have been isolated from the root bark of *Morus alba* L. (Moraceae). One is morusin, a prenylated flavone with two prenyl units, an unmodified one at position 3 and one forming an additional dimethylpyrane ring at position 8 (Fig 1). Some flavonoids that inhibit cyclooxygenase (COX)-2 activity have been studied and these all possessed a C-3 prenyl residue in their structure [2]. An investigation of the cyclization of a prenyl group attached at C-8 or C-6 found that cyclization reduced the cytotoxicity of the compounds studied but not their anti-inflammatory activity [5]. Morusin showed in previous *in vivo* studies beneficial effect on chemically induced acute bronchitis [6] and glomerulonephritis [7]. It also exerted neuroprotective effect associated with reduction of oxidative stress in brain [8].

Several *in vitro* studies describe the anti-inflammatory and cytoprotective properties of its structural derivative cudraflavone B. This flavone is able to attenuate the lipopolysaccharide (LPS)-stimulated secretion of TNF-α and the translocation of nuclear factor (NF)-κB, and inhibit the degradation of IκB, and the expression of COX-2 in macrophages [9, 10]. Hepatoprotective [11] and neuroprotective effects [12] of cudraflavone B on chemically induced cell damage have been ascribed to antioxidant activity. However, its treatment increased production of reactive oxygen species (ROS) [10]. All of these activities may be dose-dependent and could play important roles in the treatment of chronic inflammatory diseases. The 2,4,6-trinitrobenzene sulfonic acid (TNBS)-induced model of colitis in rats has been established to investigate the activity of morusin *in vivo*. Three different doses of morusin were administered to rats to observe its effectivity and involvement in the inhibition of inflammatory processes and the destruction of tissue and to compare its activity with that of the drug sulfasalazine (SAS) used clinically. For graphical summary of this study see S1 Fig.

## Materials and methods

### Plant material

The roots of *M*. *alba* were collected on the ground of the University of Veterinary and Pharmaceutical Sciences Brno, Czech Republic in April 2011. The plant material was identified by Associate Professor K. Šmejkal. A representative specimen (No. MA-11A) has been deposited in herbarium of the Department of Natural Drugs, UVPS Brno.

### Extraction and isolation

Morusin was isolated from the chloroform extract of *M*. *alba* root bark using different chromatographic methods. Twenty-two kg of dry root bark were extracted three times in ethanol and the liquid-liquid extraction of ethanolic extract was carried out with a gain of 218 g of crude chloroform fraction. TLC was done on Merck aluminum foils with silica gel 60 F254 (20 × 20 cm, 200 μm), further separations were performed using Merck silica gel for column chromatography (40–63 μm, Darmstadt, Germany). Chloroform fraction was repeatedly chromatographed over a silica gel column using mixtures of benzene/CHCl_3_/MeOH with increasing polarity to afford 42 fractions. The fraction MA2-III (28.7 g) was chosen for the final separation and divided into 11 sub-fractions. The 10 g of sub-fraction MA2-III-C-4 was purified using reverse-phase preparative HPLC (Ascentis^®^ RP-Amide, 5 μm, 250 mm × 10 mm, Supelco; Dionex Ultimate 3000 UHPLC, Thermo Scientific, Waltham, USA). Gradient elution employed 0.2% HCOOH and MeCN, the initial composition of 80% MeCN increased to final 100% MeCN after 30 min. Method was performed at a flow rate of 5 mL/min, detection wavelength 254 nm, injection volume 20 μL, and column temperature 40°C. Collecting the fraction with a HPLC tR 15.50–18.50 has led to obtaining of yellow amorphous powder with the total weight of 2,400 mg. The purity of isolated compound was determined to be nearly 99% using HPLC DAD analysis (HPLC Agilent 1100 Series with DAD UV/Vis, Agilent Technologies, Santa Clara, USA) with an analytical column Ascentis^®^ Express RP-Amide 2,7 μm, 150 mm × 4,6 mm, Supelco, Bellefonte, USA. The structure of morusin was characterized by UV spectrum (UV-Vis spectrometer Lambda 25, PerkinElmer, Waltham, USA), IR spectrum (Nicolet Impact 400D FT-IR spectrometer, Thermo Nicolet Corporation, Waltham, USA) and NMR spectra were measured in DMSO-*d*_*6*_ and recorded using a Bruker Avance 300 spectrometer at frequencies 300.13 MHz ^1^H and 75.48 MHz ^13^C (Bruker, Billerica, USA). Its identity was confirmed by comparing with the spectroscopic data of morusin isolated previously [13]. For 1D and 2D NMR spectra see Supplemental Data (S2–S9 Figs).

### Experimental animals

Male Wistar rats (180–220 g) were supplied by the Laboratory Animal Breeding and Experimental Facility of Masaryk University (Brno, Czech Republic). They were kept under standard conditions (22 ± 2°C, 50 ± 10% relative humidity), alternating 12 hour light/dark cycles. The animals had access to a standard diet and water *ad libitum*. The experimental protocol was approved by the Experimental Committee for the Welfare of Experimental Animals of the University of Veterinary and Pharmaceutical Sciences Brno, Czech Republic (Approval No. 19–2015). To minimize the suffering of the laboratory animals, the number of pharmacological interventions was limited to the necessary minimum.

### Experimental design

After one week period of acclimation the rats were divided into six groups (*n* = 8). All of the animals were fasted for 24 h prior to the induction of colitis. Colitis was induced by a single dose of TNBS (50 mg/kg; Sigma-Aldrich, Steinheim, Germany) dissolved in ethanol (50% v/v; total instilled volume: 1 mL/kg of rat body weight). TNBS was administered rectally using a rubber catheter inserted 8 cm proximal to the anus under light isoflurane anesthesia [14]. The rats were kept in a head-down position until they recovered from the anesthesia to prevent leakage of the instillation. The intact group received 0.9% saline instead of TNBS solution. Administration of the test compounds began one day after the induction of colitis and continued every 24 hours for five consecutive days. The compounds were suspended in a 10% gel of polyvinylpyrrolidone (PVP K30; Sigma-Aldrich) and were administered by gastric gavage at bolus doses of 12.5, 25, or 50 mg/kg of morusin and 50 mg/kg of sulfasalazine (Sigma-Aldrich). Rats in the untreated group received the vehicle (PVP K30) only. The animals were killed on the seventh day of the experiment by using an overdose of the veterinary euthanasia drug T61 (Intervet International B. V., Boxmeer, Netherlands).

### Macroscopic and microscopic evaluation

After dissection, the colon was removed, cut longitudinally, cleaned with cold saline (0.9%), measured, and weighed. Each colon was scored for macroscopically visible damage according to the criteria of Minaiyan et al. (2014) with the following modifications: no ulcerations (0 points), hyperemia only (1 point), mild mucosal edema (2 points), moderate edema with erosions (3 points), severe ulceration < 5 mm (4 points), severe ulceration > 5 mm (5 points). Samples about 0.5 cm long for histological analysis were obtained from the area of colon with visible ulceration or inflammation; when no grossly visible inflammation was present, the samples were excised from the region 1 cm proximal to the anus [14]. Tissue samples were fixed in 10% neutral buffered formalin and then embedded in paraffin. Three micrometer thick sections were stained with haematoxylin-eosin. The histological damage was scored by a veterinary pathologist and graded 0–3 for the severity of inflammation and infiltration of immune cells, 0–3 for the extent of inflammation (mucosa, submucosa, transmural layers), and 0–4 for crypt damage. The total histological score was the sum of all of the parameters evaluated [15].

### Preparation of colonic tissue homogenizates

Frozen colonic tissue was homogenized in lysis buffer [50 mM Tris-HCl (pH 7.5), 1 mM EGTA, 1 mM EDTA, 1 mM sodium orthovanadate, 50 mM sodium fluoride, 5 mM sodium pyrophosphate, 0.27 M saccharose] with protease inhibitors (Roche, Mannheim, Germany) as previously described [16]. The protein concentration was measured using a Bradford’s method assay kit according to the manufacturer’s instructions (Amresco, Cleveland, USA).

### Western blot analysis

The samples were denatured in the presence of β-mercaptoethanol and SDS at 70°C for 5 min. Proteins in the amount of 120 μg were separated onto 12% and 15% SDS-polyacrylamide gel, blotted to a polyvinylidene fluoride (PVDF) membrane with 0.2 μm pores (Bio-Rad, Hercules, USA), and then blocked with 5% bovine serum albumin (BSA) (SERVA, Heidelberg, Germany) in TBST buffer [10 mM Tris-HCl (pH 7.5), 150 mM sodium chloride, 0.1% (v/v) Tween 20] for 1h. The membrane was incubated with the primary antibody [rabbit anti-IL-1β 1:2500 (AbCam, Cambridge, UK; product No. ab9722), mouse anti-CAT 1:1000 (Sigma-Aldrich; product No. C0979), rabbit anti-SOD2 1:1000 (Sigma-Aldrich; product No. HPA001814), mouse anti-TGF-β1 1:2000 (AbCam; product No. ab64715), rabbit anti-TNF-α 1:1000 (AbCam; product No. ab6671), or mouse anti-β-actin 1:5000 (AbCam; product No. ab8226)] at 4°C overnight followed by washing and incubation with the secondary antibody [anti-mouse IgG (Sigma-Aldrich; product No. A0168) or anti-rabbit IgG (Sigma-Aldrich; product No. A0545) at a dilution of 1:2000] at room temperature for 1 h. Bands were visualized using a chemiluminescent kit (Bio-Rad) and a PXi Syngene Chemiluminescent Imaging System (Syngene, Cambridge, UK) and quantified by optical densitometry (GeneTools Software 4.03, Syngene).

### Zymography

Zymography was used to evaluate the activity of matrix metalloproteinases (MMP) 2 and 9. Twenty micrograms of native proteins was loaded onto 10% SDS-polyacrylamide gel impregnated with 0.1% gelatin. After separation, the gel was washed twice in 2.5% (v/v) Triton X-100 and subsequently incubated in the development buffer [50 mM Tris-HCl (pH 8.8), 5 mM calcium chloride, 3mM sodium azide, 0.5% (v/v) Triton X-100] for 15 min at room temperature and then overnight at 37°C. The gel was then stained with Coomasie blue for 2 h and destained until the bands were clearly visible. The intensity of the bands was evaluated by densitometric analysis using Gene Tools Software 4.03 (Syngene). Results were normalized to a standard control [1% fetal bovine serum (FBS), Sigma-Aldrich].

### Statistical analysis

All results are expressed as the mean with error bars representing SEM. Parametric data were analyzed using one-way ANOVA followed by Tukey’s *post hoc* test. Mann-Whitney test was performed to evaluate the score of macroscopic damage and histological score. GraphPad Prism 6.01 software (GraphPad Software, San Diego, USA) was used for the analysis. Values of *p* < 0.05 were considered to be statistically significant.

## Results

### Morusin reduces macroscopic and microscopic damage

**Fig 2:**
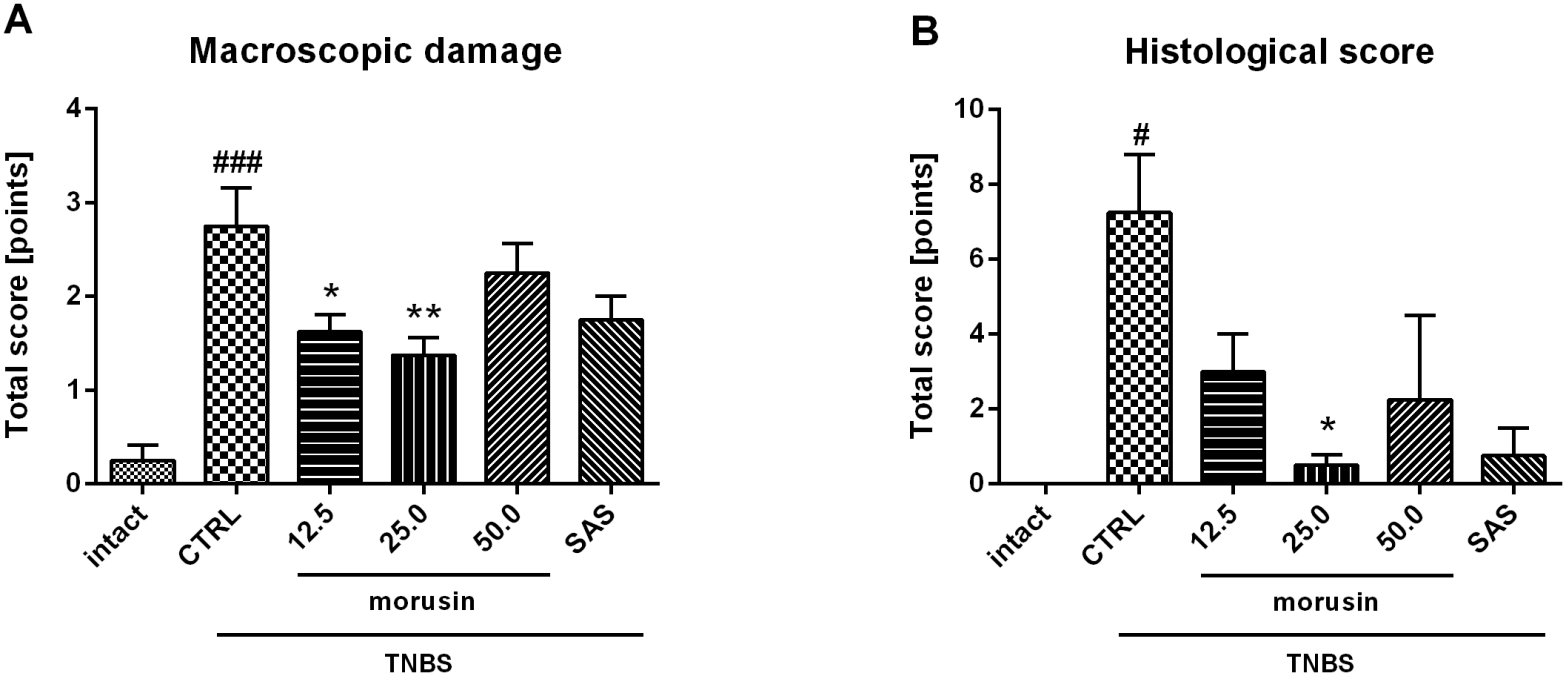
Effect of different doses of morusin (in mg/kg) on TNBS-induced colitis in rats, compared to the intact group, the untreated group (CTRL), and the group treated with sulfasalazine 50 mg/kg (SAS). (A) Score of macroscopically visible damage; (B) Histological assessment. The scoring system and parameters evaluated are described in Materials and methods. The results are expressed as the mean, with error bars representing SEM. CTRL vs. intact group: # *p* < 0.05, #### *p* < 0.0001; CTRL vs. treated groups: * *p* < 0.05, ** *p* < 0.01.

TNBS-induced colitis was characterized by transmural inflammation of the colon with signs of hyperemia, mucosal edema, and ulcerations. Adhesions to adjacent organs were visible in the untreated group. A 5-day treatment with morusin *via* gavage at doses of 12.5 mg/kg (*p* < 0.05) and 25 mg/kg (*p* < 0.01) significantly reduced the severity of colonic damage, by about 41% and 50% respectively, compared to the untreated animals (Fig 2A, Table 1). No significant reduction in the score was observed after the administration of 50 mg/kg of morusin; the animals in this group showed extensive intestinal damage. The animals treated by sulfasalazine showed non-significant reduction of score (*p* = 0.0587). The weight/length ratio of colon did not differ among the groups (S10 Fig).

**Table 1:** Score of macroscopically visible damage; Histological assessment.

	Macroscopic damage	Histological score			
	mean ± SEM	Severity of inflammation and infiltration of immune cells (mean ± SEM)	Inflammation extent (mean ± SEM)	Crypt damage (mean ± SEM)	Total (mean ± SEM)
Intact	0.25 ± 0.15	0.00 ± 0.00	0.00 ± 0.00	0.00 ± 0.00	0.00 ± 0.00
CTRL	2.75 ± 0.39^###^	2.25 ± 0.48^#^	2.25 ± 0.48^#^	2.75 ± 0.63^#^	7.25 ± 1.55^#^
Morusin 12.5	1.63 ± 0.17*	1.25 ± 0.25	1.50 ± 0.50	0.25 ± 0.25	3.00 ± 1.00
Morusin 25.0	1.38 ± 0.17**	0.50 ± 0.29	0.00 ± 0.00*	0.00 ± 0.00*	0.50 ± 0.29*
Morusin 50.0	2.25 ± 0.29	0.75 ± 0.75	0.75 ± 0.75	0.75 ± 0.75	2.25 ± 2.25
SAS	1.75 ± 0.23	0.25 ± 0.25	0.50 ± 0.50	0.00 ± 0.00*	0.75 ± 0.75

Effect of different doses of morusin (in mg/kg) on TNBS-induced colitis in rats, compared to the intact group, the untreated group (CTRL), and the group treated with sulfasalazine 50 mg/kg (SAS). The scoring system and the parameters evaluated are described in Materials and methods. CTRL vs. intact group:

^#^ *p* < 0.05,

^###^ *p* < 0.001;

CTRL vs. treated groups:

* *p* < 0.05,

** *p* < 0.01.

**Fig 3:**
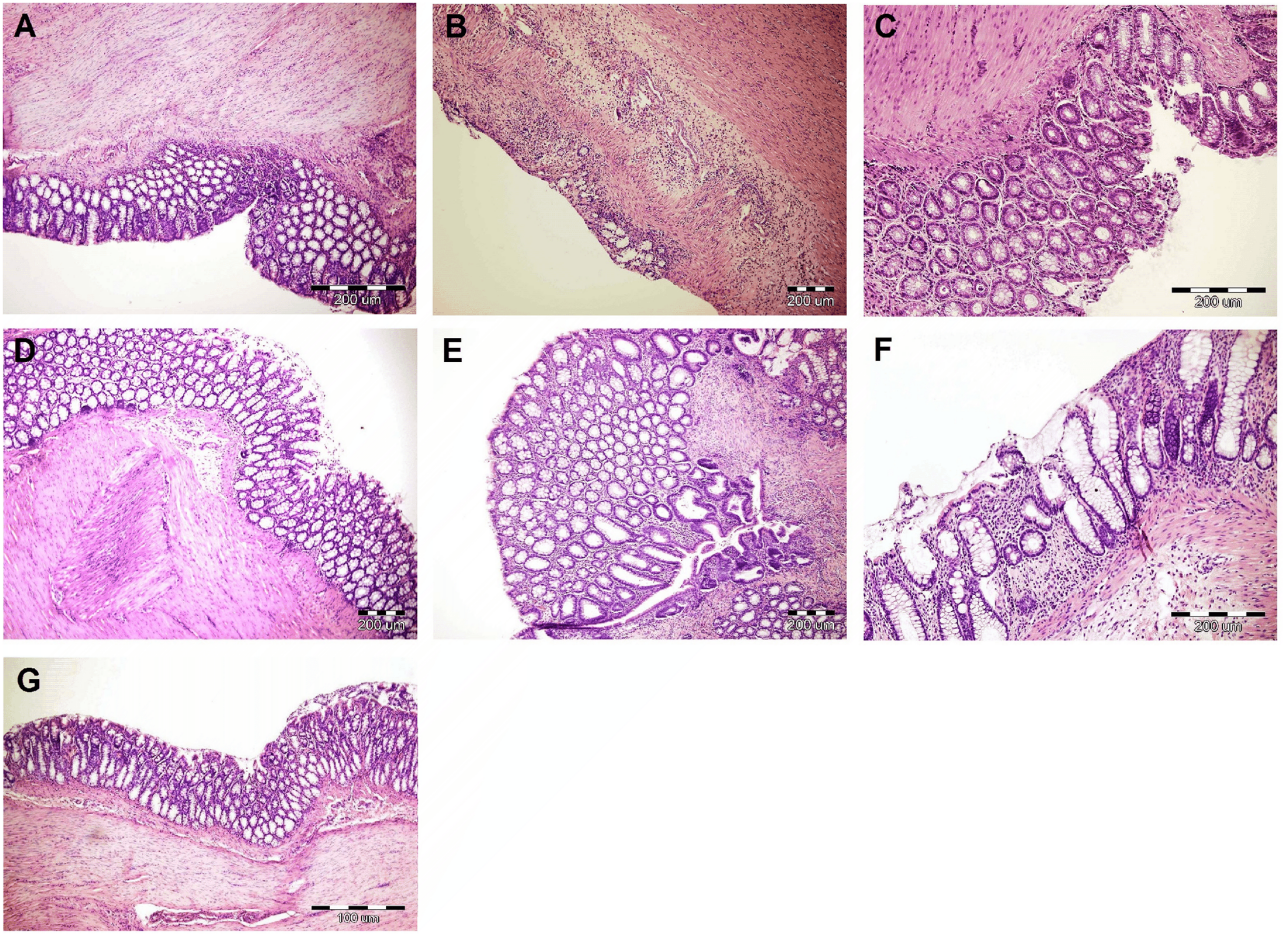
Histological findings (H&E). (A) Colon from the intact group, without induced colitis. (B) Colon after a single administration of TNBS, untreated group; tissue injury is characterized by severe crypt damage, inflammatory cell infiltration, and inflammation affecting transmural layers. Colon after induction of colitis and 5-day treatment with morusin at doses of 12.5 mg/kg (C) and 25 mg/kg (D) showing reduced extent of inflammation, infiltration and crypt damage. The 50 mg/kg dose of morusin displayed the greatest variability in tissue damage: the sample with the lowest (E) and the highest microscopic scores (F). Colon after induction of colitis and 5-day treatment with sulfasalazine 50 mg/kg (G).

Histological evaluation (Fig 2B, Table 1) of the test animals showed moderate to severe inflammation affecting the mucosa, submucosa or transmural layers, with greater infiltration of the immune cells and significant crypt damage (Fig 3B), as compared to the intact group (Fig 3A). Morusin reduced the extent of inflammation, infiltration, and crypt damage in a dose-dependent manner (Fig 3C and 3D). The microscopic damage score was reduced at the dose of 12.5 mg/kg (*p* = 0.0571) and after sulfasalazine treatment (*p* = 0.0571; Fig 3G) and significantly reduced at the dose of 25 mg/kg (*p* < 0.05). Consistent with the macroscopic evaluation, the damage score was the most variable in the group treated with the dose of 50 mg/kg (Fig 3E and 3F).

### Effect of morusin on protein expression

**Fig 4:**
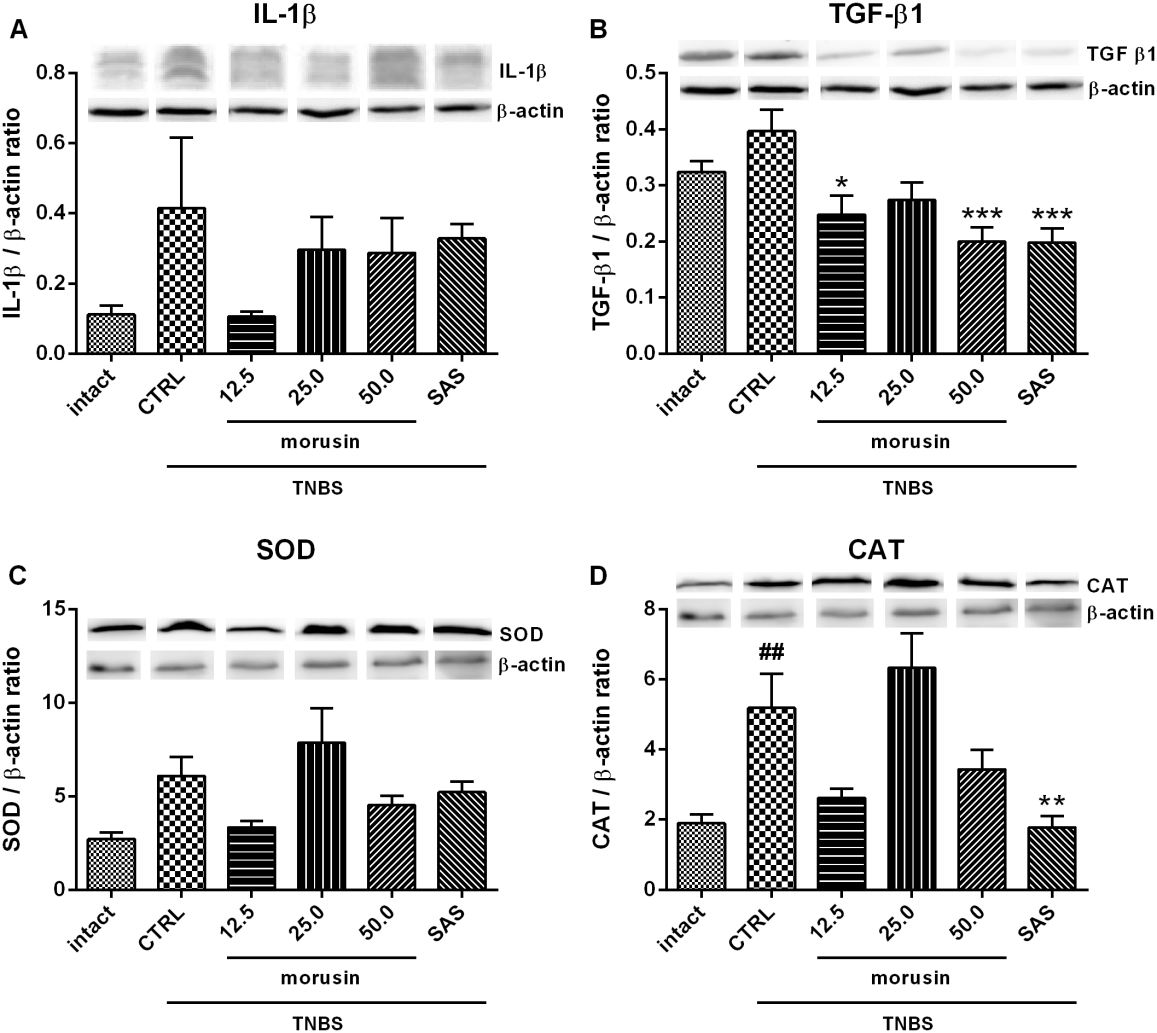
Effect of a 5-day treatment with morusin (in mg/kg) on (A) interleukin 1β, (B) transforming growth factor β1, (C) superoxide dismutase and (D) catalase levels in TNBS-induced colitis, compared to the intact group, the untreated group (CTRL), and the group treated with sulfasalazine 50 mg/kg (SAS). Values were obtained from Western blot analysis, quantified by densitometry, and normalized to the β-actin level, as described in Materials and methods. Representative blots are shown. The results are expressed as the mean, with error bars representing SEM. CTRL vs. intact group: ## *p* < 0.01; CTRL vs. treated groups: * *p* < 0.05, ** *p* < 0.01, *** *p* < 0.001.

As shown in Fig 4A, western blot analysis did not reveal significant differences in the level of IL-1β among the groups, although IL-1β was elevated after the administration of TNBS and slightly reduced after treatment with morusin. The 12.5 mg/kg dose of morusin was most effective in preventing an increase in this pro-inflammatory cytokine.

The highest level of profibrogenic factor TGF-β1 was found in untreated rats (Fig 4B). Morusin reduced its expression at all of the doses tested, significantly at 12.5 (*p* < 0.05) and 50 mg/kg (*p* < 0.001), as well as sulfasalazine (*p* < 0.001). The expression was also reduced below the level of the intact group.

The administration of TNBS also resulted in significantly greater expression of the antioxidant enzyme CAT (*p* < 0.01 compared to the intact group; Fig 4D). A significant decrease was observed after sulfasalazine treatment (*p* < 0.01). Oral treatment with morusin reduced the levels of the enzyme at doses of 12.5 and 50 mg/kg. On the other hand, the 25 mg/kg dose caused an increase in the expression of CAT. The expression of SOD corresponded to the levels of CAT (Fig 4C).

In this model, TNBS did not influence TNF-α expression, however, morusin at the dose of 25 mg/kg non-significantly decreased its level (S11 Fig).

### Effect of morusin on MMP activity

**Fig 5:**
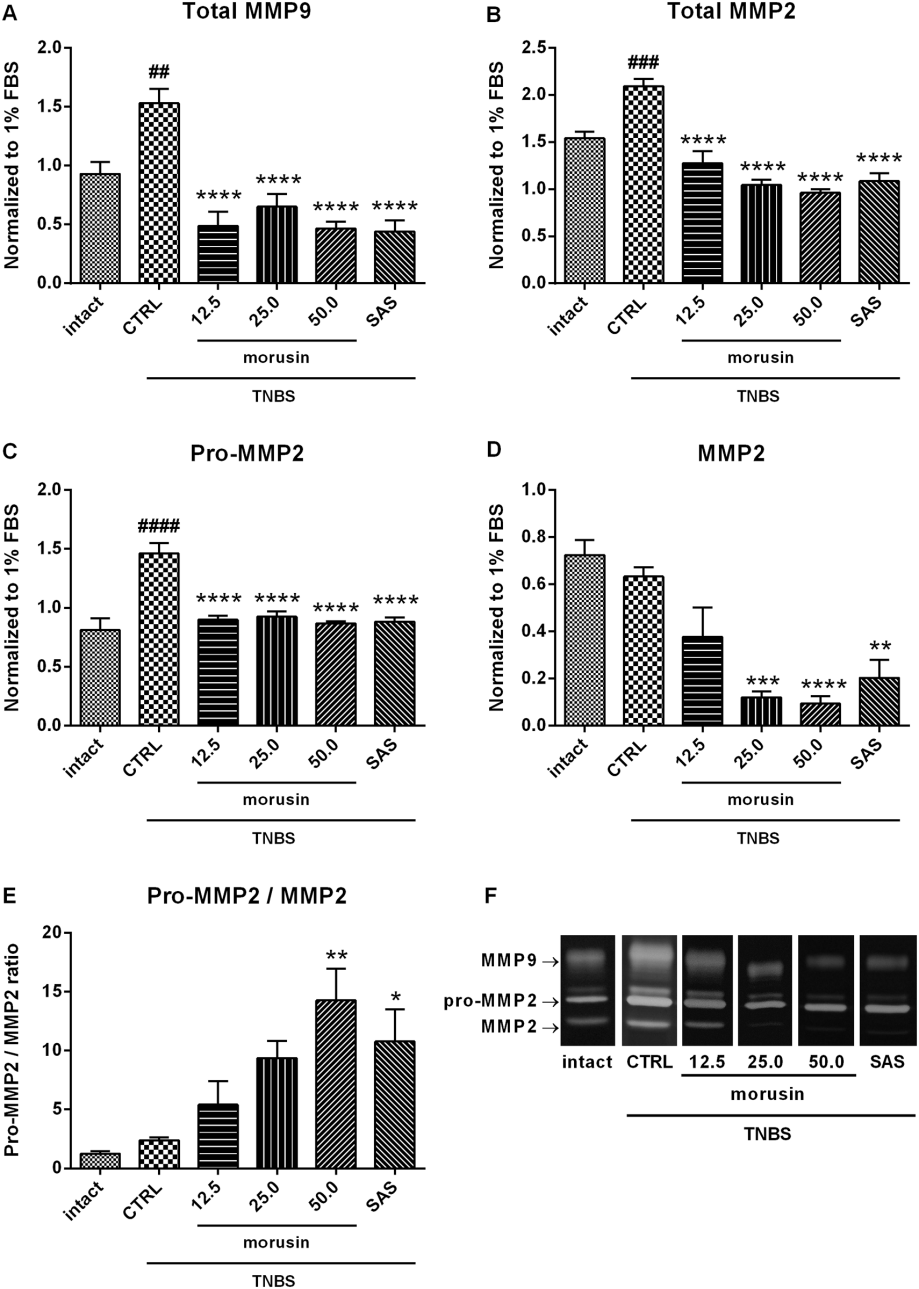
Effect of a 5-day treatment with morusin B (in mg/kg) on the activity of matrix metalloproteinases in TNBS-induced colitis, compared to the intact group, the untreated group (CTRL), and the group treated with sulfasalazine 50 mg/kg (SAS). (A) MMP9 activity, (B) total MMP2 activity, (C) activity of the pro-form of MMP2, (D) activity of the mature form of MMP2, (E) pro-MMP2/MMP2 ratio. The activity of MMPs was detected by zymography, quantified by densitometry, and normalized to a standard control (1% FBS). A representative zymogram is shown (F). The results are expressed as the mean, with error bars representing SEM. CTRL vs. intact group: ## *p* < 0.01, ### *p* < 0.001, #### *p* < 0.0001; CTRL vs. treated groups: ** *p* < 0.01, *** *p* < 0.001, **** *p* < 0.0001.

To evaluate the inflammation and tissue degradation, the activity of matrix metalloproteinases was observed (Fig 5). After administration of TNBS, the total activities of both MMP2 and MMP9 were markedly increased compared to the intact group (*p* < 0.0001 and *p* < 0.01, respectively), but no changes in the activity of the mature form of MMP2 were observed. As shown in Fig 5, all doses of the test compound significantly reduced the activity of both MMPs comparable to sulfasalazine (*p* < 0.0001). The MMP9 activity was reduced by 57–71% after treatment with morusin. The total MMP2 activity was decreased by about 39% after dose of 12.5 mg/kg and by 56% following the dose of 50 mg/kg. The activity of the mature form of MMP2 was strongly inhibited only at doses of 25 (*p* < 0.001) and 50 mg/kg (*p* < 0.0001), respectively. The ratio pro-MMP2/MMP2 increased with increasing dose of morusin, significantly so in the group treated with morusin at the dose of 50 mg/kg (*p* < 0.01) or sulfasalazine (*p* < 0.05).

## Discussion

Morusin is a flavone substituted with two prenyl chains which can distinctly modify its activity. This compound can be isolated in relatively large amounts from the root bark of *M*. *alba* by combining normal and reversed-phase chromatography, and can be obtained commercially in large amount. *Cortex mori radicis* (Sang-Bai-Pi) is a plant material used in Traditional Chinese Medicine to cure different diseases associated with inflammation; its traditional uses have been validated by many studies dealing with anti-inflammatory activity. Morusin previously showed the protective effect in different chemically induced animal models of inflammation [6, 7]. In this study, the effect of morusin on TNBS-induced colitis, an acute intestinal inflammation with predominantly Crohn’s disease-like features due to the transmural character of the inflammation, was demonstrated. According to macroscopic and histological evaluations of the colon, the greatest therapeutic effect was seen at a dose of 25 mg/kg. Whereas a lower dose also reduced the macroscopically visible damage, a higher one did not uniformly affect all of the animals in group. Studies using experimental models of bowel inflammation have usually evaluated extracts containing flavonoids, whereas analyses of the effects of the pure isolated compounds have been less frequent. Only two studies have focused on flavonoids substituted with isoprene units. Icariin, a prenylated flavonol glycoside, attenuated the progression of disease in the DSS model of inflammation [17]. In the same model, geranylated flavanones diplacone and mimulone reduced the symptoms of colitis and delayed their onset [16].

As a general marker of inflammatory response, the expression of IL-1β after morusin treatment was evaluated in this study. Its level was increased following the administration of TNBS and only the lowest dose of morusin reduced it to the level of the intact group. A previous study also showed that the structural derivative of morusin, cudraflavone B, was able to slightly reduce an elevated level of IL-1β in the presence of LPS in THP-1-derived macrophages *in vitro* [18].

The antioxidant activity of potential drugs is substantiated in IBD therapy because the oxidative damage to tissue caused by reactive oxygen species (ROS) is involved in the pathogenesis of inflammatory disorders [19]. Some of previous studies ascribed the cytoprotective effect to possible antioxidant properties of structurally similar cudraflavone B. An et al. (2006) showed a significant hepatoprotective effect against tacrine-induced cytotoxicity in human liver hepatocellular carcinoma cell line Hep G2, for which toxicity involves ROS and lipid peroxidation [11]. Lee et al. (2014) described inhibition of ROS and a neuroprotective effect against glutamate-induced neurotoxicity in mouse hippocampal HT22 cells through Nrf2 (nuclear factor-E2-related factor 2) [12]. On the other hand, Hošek et al. (2013) have reported, that cudraflavone B increased the production of ROS in the murine macrophage cell line J774.A1 [10]. To observe the antioxidant capacity in intestinal tissue, the expression of enzymes involved in the conversion of ROS (SOD, CAT), was evaluated in this study. Their expression and activity depend on the amounts of the substrates superoxide and hydrogen peroxide generated by phagocytes in inflamed tissue. This is confirmed by the higher levels of both of the enzymes evaluated in this study. Lower levels of CAT and SOD were achieved after morusin treatment at doses of 12.5 or 50 mg/kg and could imply a low level of oxidative stress. However, at a dose of 25 mg/kg the level of antioxidant enzymes was markedly increased. Such a stimulating effect on the SOD activity has previously been described for other flavonoids, such as tea catechins [20] or amentoflavone [21] with different experimental colitis models.

Intestinal fibrosis resulting from chronic transmural inflammation is a common and severe complication of IBD, especially Crohn’s disease [22]. An important profibrogenic factor, TGF-β1, is a cytokine expressed in the inflammatory condition in response to injury and related to the initiation of wound healing [23]. An increased level of it after TNBS treatment in this study may indicate the onset of development of fibrosis. Greater production of colonic TGF-β had previously been observed in both acute [24] and chronic model of TNBS-induced colitis [25]. Like sulfasalazine, morusin was able to decrease the level of TGF-β1. This reduction is consistent with the histological findings, inasmuch the intestine was not affected transmurally after morusin or sulfasalazine treatment. The expression of TGF-β was also found to be inhibited after treatment of TNBS-induced colitis with *Scutellaria baicalensis* extract, which is rich in flavones such as baicalein, baicalin, and wogonin [26].

Matrix metalloproteinases (MMP) are involved in tissue remodelation as well. The shifted balance between MMPs and their tissue inhibitors may result in mucosal membrane injury, inflammation, and tissue destruction [27]. The expression and activities of MMP2 and MMP9 were increased in different models of experimental colitis [27], moreover, MMPs were overexpressed in the inflamed tissue of patients with ulcerative colitis [28]. Morusin was distinctly able to inhibit the activities of both MMP2 and MMP9 after a 5-day oral treatment. Inhibition of the activities of both MMP2 and MMP9 in TNBS-induced colitis after administration of the anti-inflammatory drugs sulfasalazine and prednisolone has also been described by Witaicenis et al. (2012) [29]. The other parameter observed in this work, the increasing ratio of pro-MMP2 to mature MMP2 after morusin treatment, is associated with significant inhibition of the mature form. Similar results have also been described after administration of the other prenylated flavonoids diplacone and mimulone in DSS-induced colitis [16]. Inhibitors of MMPs are studied intensively as new therapeutic targets with anti-inflammatory and cytostatic indications [30]. An increased risk of developing carcinoma is associated with IBD, in ulcerative colitis particularly.

In conclusion, morusin at the dose of 25 mg/kg shows a therapeutic effect similar to or greater than that of sulfasalazine (50 mg/kg), the conventional drug used in treating IBD. The interesting mechanism of action appears to be the inhibition of the activity of matrix metalloproteinases and the levels of TGF-β1. The results suggest that morusin can be considered a promising cytoprotective agent with possible targeting to prevent complications of chronic inflammation such as fibrosis or cancer. However, this study indicates that the protective effect of morusin is limited by dose, and further studies, especially long-term treatment conditions are necessary to confirm its therapeutic potential for managing chronic inflammation.

## Supporting information

S1 Fig Graphical summary of study. (TIF)

S2 Fig ^1^H-NMR spectrum of morusin. (TIF)

S3 Fig ^1^H-NMR spectrum of morusin (detail 1). (TIF)

S4 Fig ^1^H-NMR spectrum of morusin (detail 2). (TIF)

S5 Fig ^13^C-NMR spectrum of morusin. (TIF)

S6 Fig HMBC spectrum of morusin. (TIF)

S7 Fig HMBC spectrum of morusin (detail). (TIF)

S8 Fig COSY spectrum of morusin. (TIF)

S9 Fig NOESY spectrum of morusin. (TIF)

S10 Fig Effect of morusin (in mg/kg) 5-day treatment on weight/length ratio in TNBS-induced colitis, compared to the intact group, the untreated group (CTRL), and the group treated with sulfasalazine 50 mg/kg (SAS). The results are expressed as the mean, with error bars representing SEM.
(TIF)

S11 Fig Effect of morusin (in mg/kg) 5-day treatment on TNF-α in TNBS-induced colitis, compared to the intact group, the untreated group (CTRL), and the group treated with sulfasalazine 50 mg/kg (SAS). Values were obtained from Western blot analysis of pooled samples, quantified by densitometry, and normalized to the β-actin level, as described in Materials and methods. Representative blots are shown.
(TIF)

## Abbreviations

CAT: catalase
COX-2: cyclooxygenase 2
FBS: fetal bovine serum
IκB: inhibitor of κB
IBD: inflammatory bowel disease
ICAM: intercellular adhesion molecule
IL-1β: interleukin 1β
LPS: lipopolysaccharide
MMP: matrix metalloproteinase
NF-κB: nuclear factor κB
Nrf2: nuclear factor-E2-related factor 2
ROS: reactive oxygen species
SAS: sulfasalazine
SOD: superoxide dismutase
TGF-β1: transforming growth factor β1
TNBS: 2,4,6-trinitrobenzensulfonic acid
TNF-α: tumor necrosis factor α
