# Supplementary figures and images for: Prenylated flavonoid morusin protects against TNBS-induced colitis in rats
Source: PLoS One. 2017 Aug 10;12(8):e0182464. doi: 10.1371/journal.pone.0182464 (PMC5552281; doi:10.1371/journal.pone.0182464)

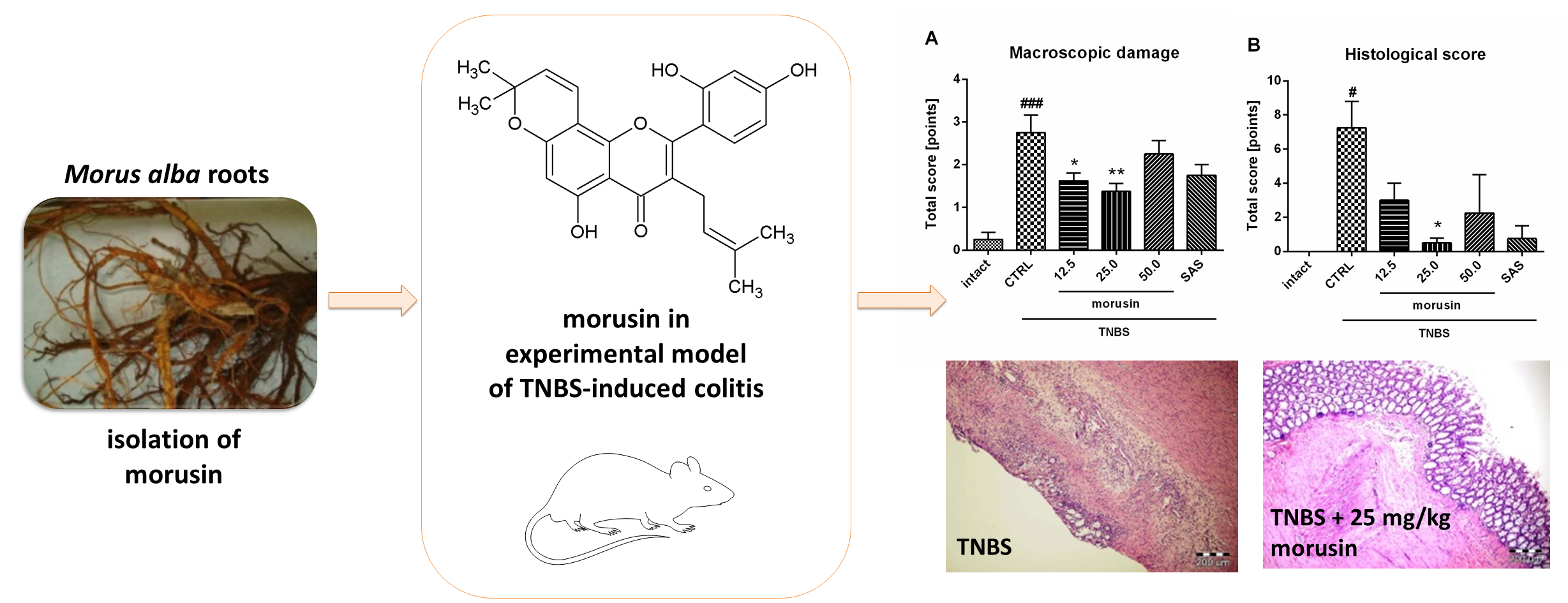

Supplement: S1 Fig — (TIF) [file pone.0182464.s001.tif]

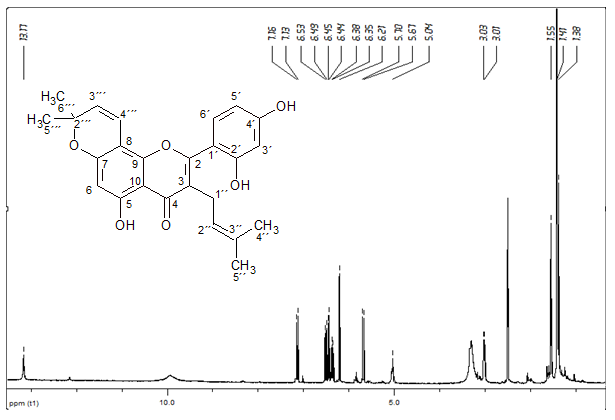

Supplement: S2 Fig — (TIF) [file pone.0182464.s002.tif]

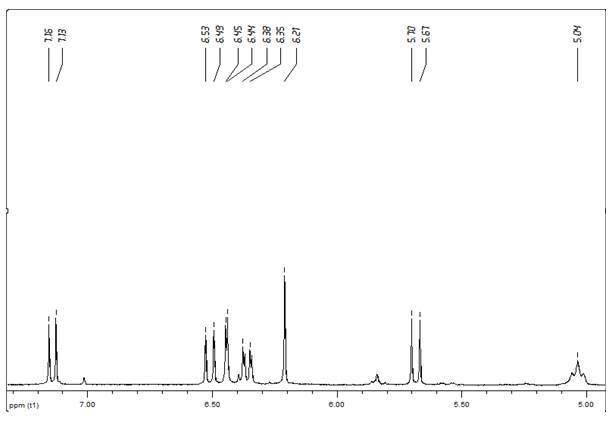

Supplement: S3 Fig — (TIF) [file pone.0182464.s003.tif]

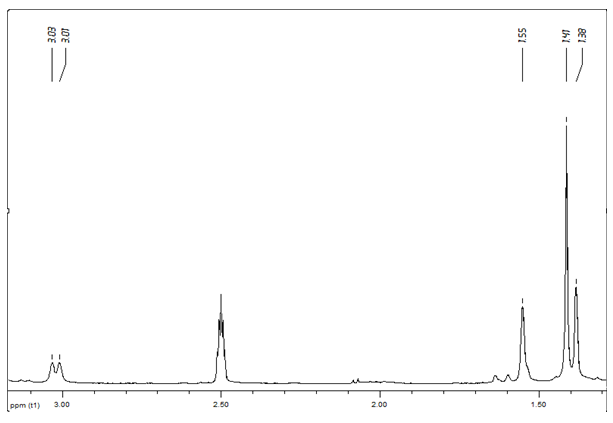

Supplement: S4 Fig — (TIF) [file pone.0182464.s004.tif]

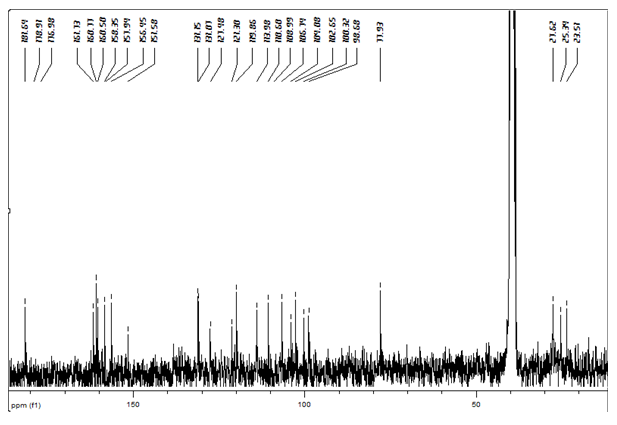

Supplement: S5 Fig — (TIF) [file pone.0182464.s005.tif]

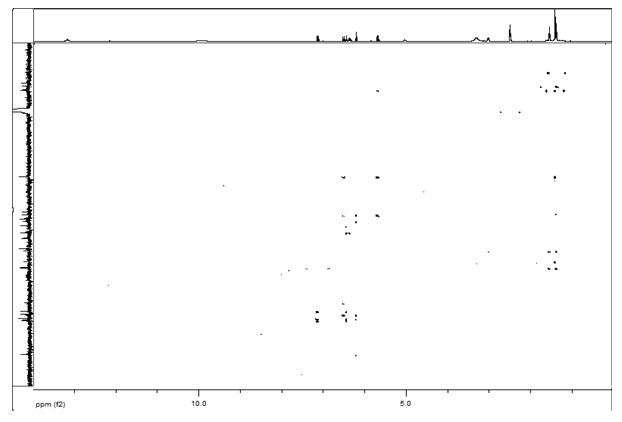

Supplement: S6 Fig — (TIF) [file pone.0182464.s006.tif]

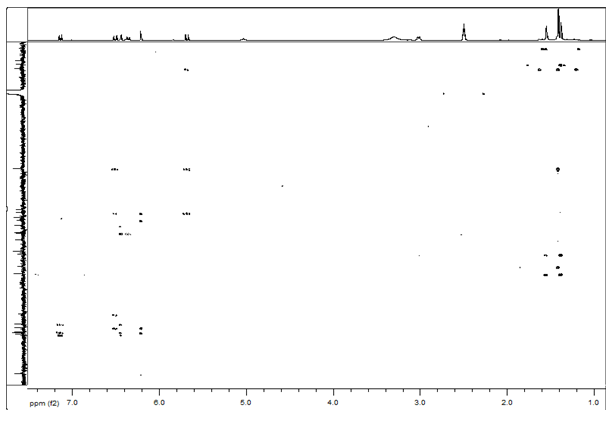

Supplement: S7 Fig — (TIF) [file pone.0182464.s007.tif]

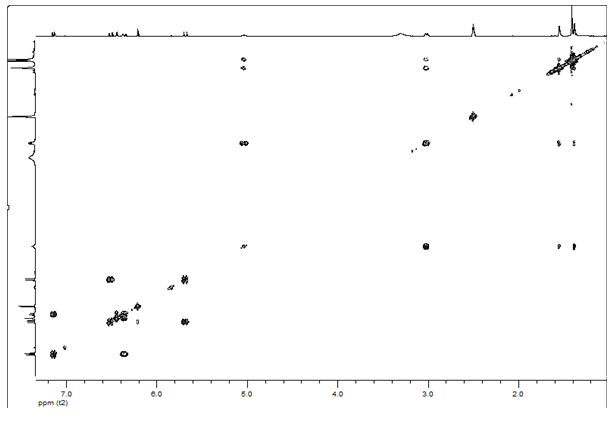

Supplement: S8 Fig — (TIF) [file pone.0182464.s008.tif]

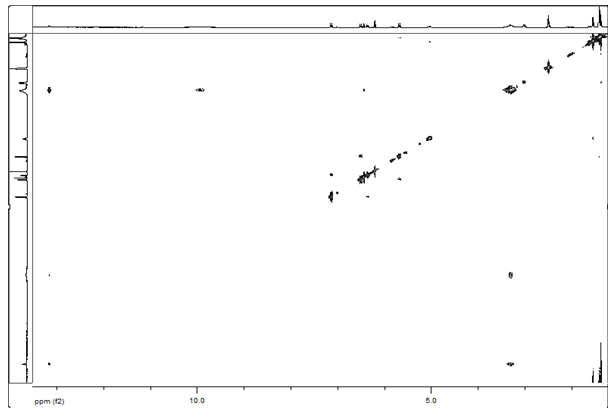

Supplement: S9 Fig — (TIF) [file pone.0182464.s009.tif]
